# Diagnosis of bladder cancer from the voided urine specimens using multi-target fluorescence *in situ* hybridization

**DOI:** 10.3892/ol.2013.1744

**Published:** 2013-12-09

**Authors:** ZUNFU KE, YUANHUA LAI, XUDONG MA, SHUHUA LIL, WENHUA HUANG

**Affiliations:** 1 Department of Pathology, The First Affiliated Hospital of Sun Yat-sen University, Guangzhou, Guangdong 510080, P.R. China; 2 Department of Vascular and Thyroid Surgery, The First Affiliated Hospital of Sun Yat-sen University, Guangzhou, Guangdong 510080, P.R. China; 3 Zhangzhou Affiliated Hospital of Fujian Medical University, Zhangzhou, Fujian 363000, P.R. China; 4 Department of Anatomy, School of Basic Medical Science, Southern Medical University, Guangzhou, Guangdong 510515, P.R. China

**Keywords:** bladder cancer, fluorescence *in situ* hybridization, molecular pathology

## Abstract

The present study aimed to evaluate the diagnostic value of chromosomal analysis by fluorescence *in situ* hybridization (FISH) for bladder cancer in light of the histological diagnosis. Several valuable observations using FISH technologies in voided urine cells were also reported. The multi-target FISH-containing probes for the centromeres of chromosomes 3, 7 and 17 and the 9p21 locus were applied to cytospin specimens prepared from voided urine. Urine samples from 53 bladder cancer patients and 30 patients with benign alterations were used for this study. The histological observations of surgical resection specimens showed that the specificity and sensitivity for the technique were 100.0 and 88.0%, respectively. Statistical analyses showed that there was no significant correlation between FISH-positive rate and the tumor stage/grade (P<0.05). However, the proportion of tumor cells with genetic abnormalities positively correlated with the tumor stage (P<0.01). Furthermore, the number of abnormal cells in muscle-invasive pT2 was significantly higher than that in non-muscle-invasive pTa, pT1 (P<0.01). Of 50 patients with bladder cancer, polysomies of chromosomes 3, 7 and 17 were detected in 84.0, 48.0 and 78.0% of cases, respectively, and loss of the 9p21 gene was detected in 80.0% of cases. In addition, the detailed results from different urine specimens showed that FISH assay was required. FISH assay for chromosomes 3, 7 and 17 and 9p21 has a high specificity and sensitivity in the detection of bladder cancer and may reduce the necessity for cytoscopy treatment.

## Introduction

Bladder carcinoma is considered to be the most common malignant tumor of the urothelium and 94% of baldder carcinomas are composed of transitional cells (1,2). Its distinct symptoms are microscopic or macroscopic hematuria and less frequent symptoms include difficulty urinating, frequent urination and therapy-resistant urinary tract infection. Therefore, it is important to detect bladder carcinoma from voided urine specimens at the earliest possible stage.

At present, urine cytology, radiographic imaging and cystoscopy are the most common methods for diagnosis and follow-up of patients with bladder cancer. Cytology is noninvasive and has a high specificity. However, its sensitivity in urinary specimens is limited as the majority of noninvasive cancers (stage pTa) are missed (3–5). Therefore, cytology alone is not reliable enough to serve as a basis for therapeutic decisions. Although full bladder ultrasound examination may show an intravesicular mass or consequential dilatation of the bladder, its sensitivity depends mainly on the quality of the apparatus and the experience of the clinician. Furthermore, it is extremely difficult for ultrasound examination to detect a tumor small in size or situated at the posterior bladder wall (6,7). In addition to discomfort and risk of patient morbidity (urinary tract infection, pain and contrast reaction), cystoscopy fails to detect flat tumors and carcinomas *in situ* in the bladder (8). Thus, a reliable and noninvasive method must be developed to detect bladder cancer in its earliest stages.

A number of previous studies have used single fluorescence *in situ* hybridization (FISH) probes to detect gene abnormalities in malignant cells; however, this may have limited sensitivity and specificity (9,10). With the progression of tumor studies, increasing numbers of new chromosomal instabilities and aneuploidies have been found in bladder cancer, particularly those involving chromosomes 1, 3, 7, 9, 11 and 17 (11–13). Therefore, hybridizing various probes for different pericentromeric and other chromosomal regions in a single step may facilitate the accurate diagnosis of bladder cancer clinically. The present study aimed to evaluate the diagnostic usefulness of multicolor FISH assay, and its sensitivity and specificity as a non-invasive method, for the diagnosis of bladder cancer.

## Materials and methods

### Patient population and samples

A total of 83 voided urine specimens (54 from males and 29 from females), obtained between June 2010 and May 2012, were collected. These included 16 samples from patients with superficial tumors (mean age, 71 years; two cases could not be evaluated by FISH evaluation) and 37 samples from patients with muscle-invasive tumors (mean age, 75 years; one case could not be evaluated by FISH evaluation). In addition, 30 patients with bladder inflammation were included in the control group (one case could not be evaluated by FISH evaluation). Voided urine specimens from all bladder cancer patients with biopsy-diagnosed bladder cancer were used as the gold standard for evidence of disease. This study was approved by the Medical Ethics Committee of Sun Yat-sen University (Guangzhou, Guangdong, China).

### FISH processing

The urine samples were collected in the morning (the first urination of the day) for FISH analysis. First, voided urine samples were centrifuged at 2,582 × g for 10 min and incubated in a hypotonic solution of potassium chloride (0.075 M). Next, the cell pellets were fixed with methanol acetic acid (2:1). Slides produced from the resuspended cells were used for the subsequent FISH assays.

Specific probe kits used in this study included CEP17, CEP3, CEP7 and 9p21 labeled with various fluorescent dyes (Beijing GP Medical Technologies, Ltd., Beijing, China). Following the guidelines of the kit, slides were developed at room temperature and then dehydrated in a series of ethanol washes (70, 85, and 100% for 2 min each). Next, a 10 μl probe mixture was added to each slide and sealed under a small glass coverslip. The target DNA and probe were codenatured at 7°C for 5 min, followed by hybridization at 42°C.

Post-hybridization washes were performed with 2X SSC for 10 min and 2X SSC/0.1% NP-40 for 5 min at 47°C. Counterstaining was performed with DAPI. Signal analysis was performed using a computer applied imaging system.

Means and three standard deviations of the percentages of nuclei with abnormal signal patterns were calculated as the cutoff values. Voided urine samples from 20 normal individuals were used to establish the cutoff values (Table I).

By definition, abnormalities, including aneusomy of locus-specific probes, chromosome monosomy and polysomy, were diagnosed only when the percentage of cells with one or three or more FISH signals exceeded the cutoff values. For each probe, 100 nuclei were studied.

### Statistical analysis

A χ^2^ test was used to analyze the correlation between FISH results and tumor stages and grades. Severity of the genetic alterations was defined as the percentage of cells with cytogenetic abnormalities according to the positive criteria of FISH. Statistical analysis of the severity of the genetic alterations among various tumor stages and grades was performed by Mann-Whitney (MW) and Kruskal-Wallis (K-W) tests, using SPSS 16.0 software (SPSS, Inc., Chicago, IL, USA).

## Results

### Genetic alterations in bladder cancer

Based on the evaluation criteria of pathological T-stage and tumor grade cancers, the 50 resected tumors with FISH examination were classified as non-muscle-invasive (pTa, pT1) in 34 cases and muscle-invasive (pT2) in 16 cases. The tumor grade was low in 37 cases and high in 13 cases.

**Figure 1:**
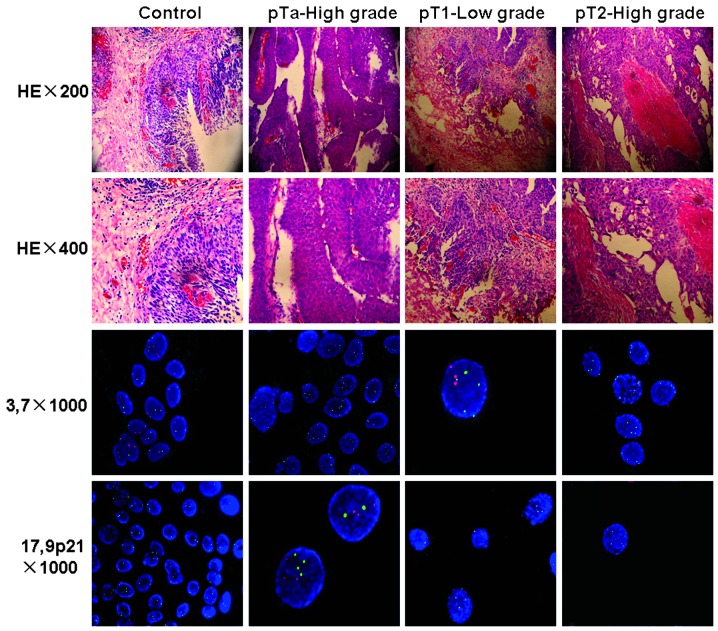
Representative images of histological findings and their corresponding FISH signal patterns for chromosomes 3, 7 and 17 and the 9p21 locus from patients with bladder inflammation (control group) and various tumor stages of bladder cancer (pTa, pT1 and pT2). Red signal represents chromosome 7 or the 9p21 locus and a green signal represents chromosomes 3 or 17. FISH, fluorescence *in situ* hybridization.

The positive rates of FISH in the urine samples were compared with the histological findings of the transurethral resection specimens. In total, 44 patients with FISH-positive results were verified histologically as having bladder cancer (Fig. 1). Negative results were obtained in six cases, from which five were proven to be carcinomas of superficial stage Ta and one was stage T2, based on the later histological findings. In the non-malignant group, 29 cases were FISH-negative and one case was not able to be detected by FISH. The detailed results are shown in Tables II and III. Altogether, based on the histological findings, the specificity and sensitivity of the FISH analysis were rated as 100 and 88%, respectively (Tables II and III).

The FISH assay showed that all cases with bladder inflammation were negative, with only chromosomes 3, 7 and 17, and the 9p21 locus, with abnormalities. Of the 44 FISH-positive transitional carcinoma cases, 8 stage Ta, 19 stage T1 and 15 stage T2 cases exhibited polysomy of chromosome 3. Polysomy of chromosomes 7 and 17 was mainly distributed in 4 and 10 stage Ta cases, 10 and 14 stage T1 cases and 10 and 15 stage T2 cases, respectively. However, in all positive samples, there was no loss of chromosomes 3, 7 and 17 detected. For 9p21, any copy number change in >6 cells was recorded as positive, which included gain of chromosomal material and heterozygous or homozygous deletions. Ten stage Ta, 19 T1 and 11 T2 cases exhibited 9p21 abnormalities (Table IV).

### Relationship between the number of abnormal cells and tumor progression

**Figure 2:**
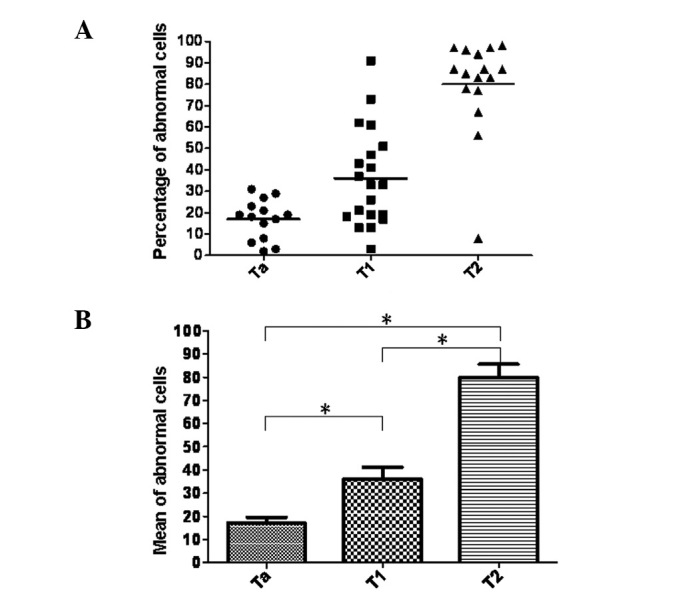
Statistical analysis of the correlation between abnormal cells and tumor stage. (A) Distribution of abnormal cells in various tumor stages. (B) The number of abnormal cells in the pT1 and pT2 (invasive) stages was higher than that in non-invasive Ta stage bladder cancer (P<0.05). The same relationship was also observed between muscle-invasive pT2 stage and non-muscle-invasive pTa, pT1 stages (P<0.01) ^*^Statistically significant difference, as stated.

**Figure 3:**
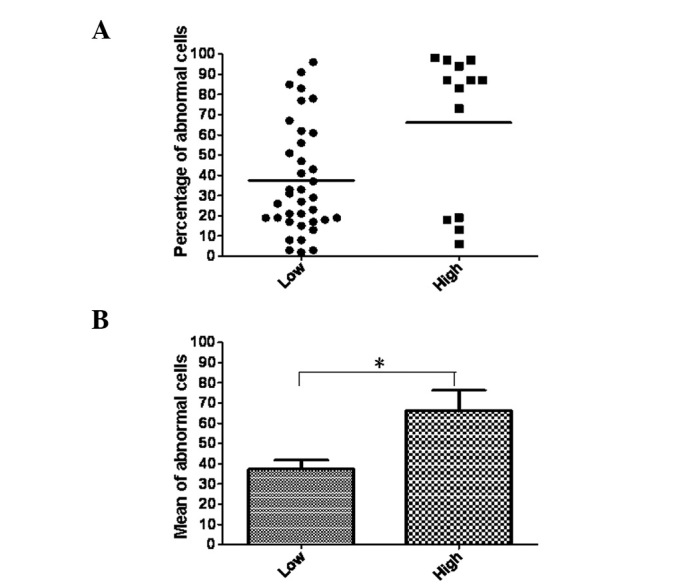
Statistical analysis of the correlation between abnormal cells and tumor grade. (A) Distribution of abnormal cells in various tumor grades. (B) The number of abnormal cells was higher in high grade than in low grade bladder cancer (P<0.01). ^*^P<0,01, vs. low grade bladder cancer.

MW and K-W tests showed a significant correlation between tumor progression and the number of abnormal cells with cytogenetic alterations. Namely, the number of abnormal cells defined by FISH testing was significantly higher in T1 and T2 (invasive) bladder carcinoma cases than in the superficial, noninvasive Ta stage bladder carcinomas (P<0.05). In addition, a significant difference was found between the number of abnormal cells in muscle-invasive and non-muscle-invasive bladder cancer (P<0.01) (Fig. 2). Furthermore, there was a positive correlation between the frequency of abnormal cells and the tumor grade and a significant difference was indicated by the MW test comparing low grade and high grade tumors (P<0.01) (Fig. 3).

### Various factors affecting FISH evaluation

**Figure 4:**
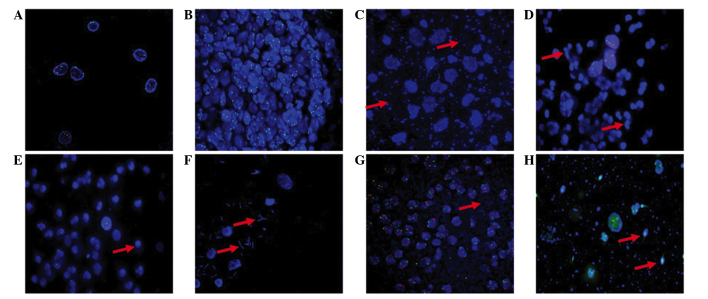
Various signal patterns were observed in the urine samples. (A) Reference urine sample. Samples in which the following observations were noted: (B) Cell density was too high, (C) large volume of mucus among cells, (D) an excessive number of neutrophils, (E) an excessive number of lymphocytes, (F) numerous bacilli among epithelial cells, (G) numerous cocci among epithelial cells and (H) an excessive number of sperm among epithelial cells.

FISH evaluation was affected by various factors, particularly by specific characteristics of the urine sample. Fig. 4A shows an optimal cytospin specimen in which the cells are evenly spread and the contours of the cell nucleus are extremely clear. However, in Fig. 4B, the high cell density makes it difficult to discern cell contours, which affect the positive rate of FISH. Occasionally, a high proportion of mucus also leads to the failure of pepsin function so that the probes for chromosomes 3, 7 17 and 9p21 fail to hybridize with the corresponding complementary target sequences (Fig 4C). In patients with severe inflammation, numerous lymphocytes and neutrophils may cover the epithelial cells, resulting in errors in signal dot counting (Fig. 4D and E). Similarly, bacilli, cocci and sperm may make the background too concentrated, which can affect the appearance of the green signal (Figs. 4F–H).

## Discussion

Bladder cancer is one of the most common malignancies and represents the 13th most common cause of all cancer mortalities worldwide (14). The majority of patients with bladder cancer produce hematuria whilst the other 10% exhibit asymptomatic microscopic hematuria, which is positive for the morphological abnormality of urine cytology (15). Thus, the main method for diagnosis of bladder cancer is a combination of cystoscopy, biopsy and voided urine cytology. Although urinary cytology is non-invasive and highly sensitive for cases of differentiated tumors, it also has disadvantages, for example being examination-dependent (16). Furthermore, cystoscopy is an invasive diagnostic intervention accompanied by discomfort and potential complications. Therefore, urine-based marker systems are important for the diagnosis and treatment of bladder cancer.

The FISH technique was approved by the FDA for the detection of urothelial carcinomas in July 2001 (17). Following this, a number of studies have shown promising results for FISH technology (18,19). In a comparative study of FISH with the diagnostic markers, BTA Stat, hemoglobin strips and telomerase, FISH exhibited the highest sensitivity (81% compared with 78, 74 and 46% for BTA Stat*,* hemoglobin strips and telomerase, respectively) and the highest specificity (96%) (17). The present study found the specificity of the method to be 100% and the sensitivity to be 87%. It was noted that 93.8% of the invasive bladder tumors were detected (excluding the cases with FISH results that could not be evaluated). However, FISH results misidentified five cases with non-muscle-invasive (pTa, pT1) bladder cancer and one case with muscle-invasive (pT2) bladder cancer. Possible reasons for these false-negative FISH results include a low tumor burden or tumor cells that were not readily excreted into the urine, particularly in non-muscle-invasive stages (20). In addition, these results may be associated with combination pattern of FISH probes, including chromosomes 3, 7, 9 and 17, which are the most common abnormalities found in urothelial carcinoma (21). These results showed that the FISH assay significantly increases the sensitivity and specificity of the diagnosis of bladder cancer, as described in previous studies (22,23).

Waldman *et al* have shown that the severity of chromosomal aberrations is closely associated with high-grade and high-stage bladder carcinoma, aggressive behavior and a significant reduction in the progression-free interval (24). Results of the present study indicate that the severity of genetic alterations is significantly higher in high-grade and high-stage carcinomas, since the frequency of abnormal cells matching the positive criteria of FISH analysis is positively correlated with tumor invasiveness (stages pTa-pT1 and pT2) and histological grade (low-high). It has been suggested that a tetraploidization process may lead to the majority of the chromosomal aberrations detected by FISH (25). By contrast, aneusomy of chromosomes 7, 9, and 17 was hypothesized to be predictive of tumor recurrence in a separate study (26). The present study also showed that the positive rate for polyploidy of chromosomes 3, 7 and 17 in invasive stages (pT1 and pT2) was significantly higher than that in the superficial stage (pTa). This indicated that the existence of multiple cytogenetic changes was correspondingly associated with the aggression of bladder cancer.

During FISH analysis in the present study, the final diagnostic evaluation was influenced by various factors, particularly characteristics of the urine sample. These included the chemical features of the urine, neutrophil or lymphocyte infiltration, bacteriuria, sperm and the time point of urination. If excessive grume is found in the urine, the assimilation time of pepsin can be extended. In addition, increasing the washing frequency with 0.2X SSC may clear away adhesive neutrophils and lymphocytes which make evaluation extremely difficult through their association with epithelial cells. In general, instructing patients with bacteriuria to take antibacterials three days before FISH is important as bacteria may adhere to the epithelial cells and make correct detection impossible, particularly in 9p21 signals. Furthermore, the experience of the doctor may also be critical. It is important not to regard the multiple signals of overlapping cell as a chromosome multiplication. To avoid this, it is imperative that the channel of the nuclear staining is always carefully examined using DAPI.

At present, there is no effective method to replace cystoscopy, however, FISH may represent an additional diagnostic tool providing help in unclear cases. Furthermore, FISH has advantages over urine cytology in detecting bladder cancer, for example a higher specificity and sensitivity, which may play a vital role in the initial diagnosis and treatment of patients with bladder cancer.

## Figures and Tables

**Table I:** Defining the optimal cutoff values for FISH-positive voided urine specimens (average percentage of cells with 0, 1, 3 or more signals in 100 consecutive cells from 20 urine specimens from normal donors).

	CSP 3		CSP 7		CSP 17		9p21	
				
Number of signals	1	3 or more	1	3 or more	1	3 or more	0 or 1	3 or more
Mean	2.18	0.65	1.85	0.50	1.53	0.83	2.15	2.03
SD	1.38	0.61	1.28	0.50	1.34	0.63	1.25	1.32
Cutoff, %	6.32	2.48	5.69	2.00	5.55	2.72	5.90	5.99

FISH, fluorescence *in situ* hybridization; CSP, chromosome-specific probe; SD, standard deviation.

**Table II:** FISH results in voided bladder cancer urine specimens.

	FISH results			
		
Diagnosis type	Positive	Negative	Could not be evaluated	Total
Malignant				
Bladder cancer	44	6	3	53
Non-malignant				
Inflammation	0	29	1	30

FISH, fluorescence *in situ* hybridization.

**Table III:** Multiprobe FISH results in voided bladder cancer urine specimens of various stages and grades.

	FISH results		
		
Bladder cancer	n/N	%	χ^2^ test P-value
Stage			>0.05
Non-muscle-invasive (pTa, pT1)	29/34	85.3	
Muscle-invasive (pT2)	15/16	93.8	
Grade			>0.05
Low	32/37	86.5	
High	12/13	92.3	
Overall sensitivity	44/50	88.0	

FISH, fluorescence *in situ* hybridization; n, number of cases exhibiting genetic abnormalities; N, total number of cases.

**Table IV:** Comparison between individual FISH probes and the combined probe in 79 voided urine specimens.

Combined	Centromeric probe (polysomy)			Homozygous deletion of 9p21	Deletion of 9p21

	3	7	17		
Inflammation (n=29)	2 (6.0)	1 (3.5)	3 (10.4)	1 (3.5)	0 (0.0)
pTa (n=14)	8 (57.1)	4 (28.6)	10 (71.4)	10 (71.4)	10 (71.4)
pT1 (n=20)	19 (95.0)	10 (50.0)	14 (70.0)	19 (95.0)	19 (95.0)
pT2 (n=16)	15 (63.4)	10 (62.5)	15 (93.8)	11 (68.8)	15 (93.8)

FISH, fluorescence *in situ* hybridization.
